# Three discipline collaborative radiation therapy (3DCRT) special debate: I would treat all early‐stage NSCLC patients with SBRT

**DOI:** 10.1002/acm2.12545

**Published:** 2019-02-22

**Authors:** Pranshu Mohindra, Amit Sawant, Robert J. Griffin, Narottam Lamichhane, Erina Vlashi, Meng Xu‐Welliver, Michael Dominello, Michael C. Joiner, Jay Burmeister

**Affiliations:** ^1^ Department of Radiation Oncology University of Maryland School of Medicine Baltimore MD USA; ^2^ Department of Radiation Oncology University of Arkansas for Medical Sciences Little Rock AR USA; ^3^ Department of Radiation Oncology University of California‐Los Angeles Los Angeles CA USA; ^4^ Department of Radiation Oncology The James Cancer Center Ohio State University Columbus OH USA; ^5^ Department of Oncology Wayne State University School of Medicine Detroit MI USA; ^6^ Gershenson Radiation Oncology Center Barbara Ann Karmanos Cancer Institute Detroit MI USA

**Keywords:** lung radiotherapy, NSCLC, SBRT

## 1 THREE DISCIPLINE COLLABORATIVE RADIATION THERAPY (3DCRT) DEBATE

Radiation Oncology is a highly multidisciplinary medical specialty, drawing significantly from three scientific disciplines — medicine, physics, and biology. As a result, discussion of controversies or changes in practice within radiation oncology involves input from all three disciplines. For this reason, significant effort has been expended recently to foster collaborative multidisciplinary research in radiation oncology, with substantial demonstrated benefit.^1^, ^2^ In light of these results, we endeavor here to adopt this team‐science approach to the traditional debates featured in this journal. This article represents the second in a series of special debates entitled “Three Discipline Collaborative Radiation Therapy (3DCRT)” in which each debate team will include a radiation oncologist, medical physicist, and radiobiologist. We hope that this format will not only be engaging for the readership but will also foster further collaboration in the science and clinical practice of radiation oncology.

## 2 INTRODUCTION

Stereotactic Body Radiotherapy (SBRT) has emerged as an effective treatment for early‐stage inoperable Non‐Small Cell Lung Cancer (NSCLC) patients. We currently have strong evidence for the safety and efficacy of SBRT for patients with tumors outside of the proximal tracheobronchial tree. As SBRT gains more widespread use, evidence is beginning to mount which not only supports the safety of SBRT for centrally located lesions, but also suggests that SBRT may be a viable alternative to surgery for operable patients. SBRT may offer the possibility of local control and long‐term survival similar to surgery, but with decreased procedural morbidity, therefore improved quality of life (QoL) for such patients. However, surgery currently remains the standard of care for the treatment of resectable, early‐stage NSCLC. SBRT has thus far demonstrated significant efficacy and the ability to reduce the risk of complications for some patients, but can we yet make a convincing case for SBRT as the treatment of choice for all patients with early‐stage NSCLC?

Arguing for the proposition will be Drs. Pranshu Mohindra, Amit Sawant, and Robert J. Griffin. Dr. Mohindra is a faculty radiation oncologist at the University of Maryland School of Medicine with research interests in evaluating outcomes for thoracic, gynecological, and hemato‐lymphoid malignancies through institutional and population‐based databases, use of modern radiation techniques including proton therapy and early phase clinical trials. He is the national principal investigator for the National Cancer Institute Experimental Therapeutics Clinical Trials Network phase‐I studies NCT02589522 and NCT02993146, co‐chair for the Alliance A041501A study and Alliance co‐chair for NRG LU002.

Dr. Sawant is a faculty medical physicist at the University of Maryland School of Medicine whose research interests include advanced motion management for lung SBRT, virtual bronchoscopy guided functional preservation for lung SBRT, high‐performance computing‐based treatment planning, and small animal image‐guided radiotherapy. He serves on several workgroups and committees for the AAPM, including Chair of the Workgroup for Research Funding and Vice Chair of AAPM TG264 on safe clinical implementation of real‐time MLC tracking.

Dr. Griffin is a professor of radiation biology at the University of Arkansas for Medical Sciences. His group studies the living tissue response to radiotherapy and targeted drug delivery to tumors. He served as the president of the Society for Thermal Medicine, is Vice Chair of the Science Education and Professional Development Committee, and Annual Meeting biology track chair for ASTRO. He is associate senior editor for both Technology in Cancer Research and Treatment and the International Journal of Radiation Oncology, Biology, and Physics.

Arguing against the proposition will be Drs. Narottam Lamichhane, Erina Vlashi, and Meng Xu‐Welliver. Dr. Lamichhane is an assistant professor and medical physicist in the Department of Radiation Oncology at the University of Maryland School of Medicine. He completed his therapeutic medical physics residency from the University of Miami — Miller School of Medicine. His training and research interests focus on the treatment planning, quality assurance, imaging, and experimental therapeutics.

Dr. Vlashi received her PhD in Chemistry from Purdue University, followed by postdoctoral training in cancer stem cell biology in the Department of Radiation Oncology at UCLA, where she is now an assistant professor. Dr. Vlashi's current research interests include investigating the effect of radiation therapy on cancer cell redox metabolism to identify targetable vulnerabilities.

Dr. Xu‐Welliver is currently an associate professor in radiation oncology at the Ohio State University James Cancer Center. Her clinical focus includes thoracic cancers, lymphoma and sarcoma. Her research focuses on designing rational combination therapy with radiation to improve treatment outcomes.

## 3 OPENING STATEMENTS

### 3.A Pranshu Mohindra, MD; Amit Sawant, PhD; Robert Griffin, PhD

Very few recent lung cancer therapies have had as positive an impact on public health as lung SBRT, which involves the precise administration of very high, biologically potent doses in 1–5 fractions. Surgery is the current standard‐of‐care (SOC) for operable early‐stage NSCLC patients. We argue that lung SBRT should become the new SOC for these patients. Our position is based on (a) mounting clinical evidence, (b) modern technological advances, and (c) recently improved understanding of the radiobiology of SBRT.

First, Phase III randomized controlled trials comparing SBRT vs surgery, such as the STABLE‐MATES trial in the US (NCT02468024), the stereotactic ablative radiotherapy SABR Tooth trial in the UK (ISRCTN13029788), and the US Veterans Affairs VALOR trial (NCT02984761), are still accruing or awaiting maturation. However, compelling evidence from clinical trials of various surgical techniques and retrospective meta‐analyses of SBRT studies suggests that SBRT has comparable or even superior oncological outcomes, with lower risk of toxicity compared to surgery.

In the case of surgery, current options for early‐stage NSCLC include lobectomy for patients deemed completely operable (CO) or sub‐lobar resection for those deemed marginally operable (MO). The benchmark Lung Cancer Study Group clinical study in T1N0 NSCLC patients noted that, compared to lobectomy, sub‐lobar resection had a 75% increased recurrence rate (*P* = 0.008) and a 30% increase in overall death rate (*P* = 0.08) without improvement in perioperative morbidity, mortality, or late postoperative pulmonary function.^3^ These findings indicate that MO patients, who cannot undergo lobectomy, may be strong candidates for SBRT. Whereas conventional thoracotomy for lobectomy is associated with poor postoperative quality of life (QoL); the modern surgical technique of video‐assisted thoracoscopic surgery (VATS) has shown significant reduction in postoperative pain and improved recovery in a recent randomized controlled trial.^4^ A recent Swedish nationwide cohort study demonstrated a 5‐yr overall survival (OS) of 78% with VATS.^5^

In the case of SBRT, multiple studies have shown comparable OS and superior QoL to lobectomy. Among the largest experiences of long‐term outcomes, a Japanese multi‐institutional study with 87 CO patients who refused surgery and were treated with SBRT, demonstrated 5‐yr OS of 78%.^6^ In a follow‐up update of 661 operable patients, a 3‐yr OS of 79% was noted.^7^ A pooled analysis of two randomized trials (STARS and ROSEL) showed significantly superior 3‐yr OS for SBRT compared to surgery (95% vs 79%, *P* < 0.05) with 10% vs 44% grade 3/4 treatment‐related toxicity.^8^ A comparison of pulmonary function tests has demonstrated consistent decline by 11%–17% of predicted values with surgery whereas use of SBRT resulted in a declining trend of only 6% after 22 months.^9^ From a QoL and cost perspective, analysis of patient‐reported outcomes from the ROSEL study demonstrated better global health related QoL with lower total productivity cost to society (*P* = 0.044) and lower patient‐reported hindrance in paid and unpaid work (*P* = 0.01) with the use of SBRT.^10^

Second, advances in motion management and image guidance have broadened the scope for the use of SBRT for centrally located and/or larger tumors.^11^ These include the increased use of four‐dimensional (4D) CT, in‐room image‐guidance based on cone‐beam CT, CT on‐rails, recently developed integrated MRI + radiotherapy machines, and real‐time position monitoring (optical markers, abdominal belts, surface photogrammetry). All of these have led to increased precision and accuracy and therefore increased confidence in administering more potent dosing regimens such as single‐ to three‐fraction SBRT protocols. Reduced fractionation has also benefited patients in terms of increased convenience of treatment and improved overall cost‐effectiveness.^12^

Third, there may be a biological basis that could also swing the pendulum in favor of SBRT. In an institutional analysis, SBRT treatments were noted to deliver an incidental mediastinal dose of <5 Gy of the prescription dose for the majority of patients.^13^ Yet, the 4.9% incidence of mediastinal recurrence was lower than that would be expected historically, raising the hypothesis of distant immune effects with the use of SBRT. Indeed, the possibility of tumor antigen presentation with ablative treatments and its impact on immunotherapeutic treatments is yet to be fully explored,^14^ and there are ongoing clinical trials combining SBRT with immunotherapy in lung cancer.^15^, ^16^, ^17^

Note that head‐to‐head retrospective comparisons of surgery and SBRT are limited (and arguably biased against SBRT) due to primary use of SBRT for medically inoperable patients. These patients present with existing comorbidities, confounding the survival outcome. There is also stage‐migration in favor of surgical series, which exclude patients with nodal disease in the surgical specimen. This is especially relevant for population‐based analyses which have shown conflicting results between surgery and SBRT even when using propensity score analysis.^18^, ^19^, ^20^ Indeed, one analysis of medically operable vs inoperable patients treated with SBRT has also demonstrated lower OS in the latter group.^21^ Data on an intent‐to‐treat analysis of surgery vs SBRT are also lacking.

A common rationale employed to justify surgery over SBRT is the low risk of postoperative complications including deaths with VATS surgery. Nonetheless, it is important to note that in the most recent randomized assessment from a large Dutch group, clinically relevant pain in the first 24 h was seen in 38% of the VATS patients with 9.2% (7/76) patients continuing to note moderate to severe pain even at 52‐weeks follow up. Postoperative grade 3–4 events were noted in 23.5% (24/102) patients. In comparison, in the Japanese multi‐institutional SBRT experience, grade 3 or greater complications were noted in 1.9% patients with 0.5% treatment‐related deaths.^7^

One limitation of SBRT is the lack of formal surgical nodal staging which results in inclusion of patients with micrometastatic disease, resulting in higher rates of regional recurrence in comparison to surgery.^22^ Use of modern endobronchial ultrasound and PET‐CT‐based staging may reduce this disparity. Additional research with the use of circulating tumor cell assessment in presumed early‐stage NSCLC may also provide lead‐time notice of disease recurrence or progression.^23^ Finally, with the use of modern treatment paradigms, salvage of isolated nodal failure is feasible.^24^, ^25^

On the other hand, a significant limitation of surgery is the management of patients presenting with more than one synchronous or metachronous lesion. While lobectomy may be feasible for one lesion, a second lobectomy will generally not be preferred. In contrast, recent experiences have demonstrated the safety of multiple courses of SBRT.^26^ Furthermore, for patients who are considered high risk for serious toxicity from SBRT, like patients with interstitial lung disease, the associated poor pulmonary function may also make surgery a high‐risk treatment. For such patients, using alternate approaches of hypofractionated radiation therapy with novel systemic therapy may need to be explored.

In summary, a patient who walks into a multidisciplinary thoracic oncology clinic with newly diagnosed early‐stage NSCLC has two clear options that are equally efficacious but distinct: (a) undergo an invasive procedure under general anesthesia that is highly dependent on the individual provider's skills, and may involve a short in‐patient stay followed by a few weeks of healing and a small risk of early mortality, or, (b) undergo a noninvasive, out‐patient treatment that can be completed in as few as one to five sessions, that allows the patient to continue all activities of daily living without interruption but with some risk of delayed or long‐term complications. Based on the reasoning provided above, we believe that SBRT should be the treatment of choice for all CO and MO early‐stage NSCLC patients.

### 3.B Narottam Lamichhane, PhD; Erina Vlashi, PhD; Meng Xu‐Welliver, MD, PhD

There are over 200 000 new cases of lung cancer each year^27^ and approximately 85% of these are NSCLCs. Among these, about 15% present at an early, localized stage.^28^ Surgery, in the form of lobectomy, bi‐lobectomy, or segmentectomy remains the standard of care in early‐stage (T1T2N0) NSCLC and results in 5‐yr survival rates of 60%–70%.^3^, ^29^ However, many patients are medically inoperable at the time of diagnosis due to existing comorbidities, such as chronic obstructive pulmonary disease (COPD) and cardiovascular compromise. For this group of early‐stage NSCLC patients, stereotactic body radiation therapy (SBRT) is at the forefront and has become the treatment of choice. With more advanced 4D imaging techniques, motion management, conformal treatment planning, and daily imaging guidance, SBRT is able to deliver high doses in few fractions in a highly conformal fashion providing a steep dose fall‐off outside the target.^30^, ^31^ In the medically inoperable early‐stage NSCLC setting, SBRT achieves local control rates of ~90% at 3–5 yr and overall survival (OS) of 55%–60% at 3 yr and has been established as a safe and effective alternative treatment option for this group of patients.^32^ However, due to excellent local control rates, low toxicity and the noninvasive delivery of SBRT, there has been an increased use of SBRT in early‐stage NSCLC patients who are candidates for surgery. While there seems to be no debate in the community regarding the effectiveness of SBRT in treating medically inoperable early‐stage NSCLC, the key word in the statement debated here — “I would treat all early‐stage NSCLC patients with SBRT” — is the word “all,” as this would include early‐stage NSCLC patients who are good candidates for surgery.

To date, there has been no completed prospective randomized study comparing lobectomy and SBRT in surgically operable patients. Three such trials (ROSEL, STARS, and RTOG 1021/ACOSOG Z4099) closed early due to poor accrual.^33^ Currently there are three ongoing trials designed to answer this very question, the United States STABLE‐MATES trial,^34^ the U.K. SABRTooth trial,^35^ and the Veterans Affairs VALOR trial.^36^ Outside of the prospective randomized studies, there have been numerous retrospective analyses comparing these two treatment modalities with some showing superior survival for surgery.^19^, ^37^ However, since patients who undergo SBRT have worse performance status and more medical comorbidities, it is hard to avoid bias in these comparison studies with retrospective data. In a 2016 NCDB study that included only patients free of comorbidities, 13 562 stage I lung cancer patients treated with lobectomy were compared to 1781 patients treated with SBRT; this revealed a 5‐yr OS of 59% for lobectomy vs 29% for SBRT.^18^ This is in contrast to a Dutch propensity‐ matched analysis of SABR and VATS lobectomy patients, which showed superior 3‐yr locoregional control with SABR (93.3% vs 82.6%) with no difference in distant recurrence or OS.^20^ Clearly, continued support for randomized prospective stage III studies comparing surgery vs SBRT is still very much needed, thus arguing against using SBRT for “all” early‐stage NSCLC until conclusive outcome data from clinical trials is available.

Tumor location plays an important role in deciding if a patient should be treated by surgery or radiotherapy. While tumors located in the periphery of the lung can tolerate single fraction or 3‐fraction SBRT, it is not safe to treat tumors located more centrally and close to critical organs at risk (OARs), such as the heart, major vessels and proximal airways. This brings us to the next issue with the word “all” in this statement, as delivering high single doses of radiation to central tumors (within 2 cm of the tracheobronchial tree) even with the most highly conformal technology can result in excessive toxicity,^38^ especially if the treatment is delivered in ≤3 fractions.^32^, ^38^ To mitigate toxicity, “risk‐adapted” SBRT regimes of 4–8 fractions are now being used to treat central tumors.^32^, ^39^ However, SBRT is still not recommended for “ultra‐central” lung tumors which directly touch major airways and more conventional schedules (with lower biological equivalent dose (BED) and hence lower control rate) are recommended.^32^, ^40^ Patients with centrally located tumors remain a therapeutic challenge for both surgery and radiotherapy.

As with most cancers, not all NSCLCs are created equal, even at an early stage. The two main histologic groups of adenocarcinomas and squamous cell carcinomas of the lung are not only different in their histological classification and cell of origin but also differ genetically,^41^, ^42^ metabolically,^43^ and immunologically.^44^ Therefore, the biology and radiobiological response in these subgroups is most likely different in important ways and this should not be ignored until more information is available. Conceivably some tumors would be more radioresistant than others and surgery may be more appropriate for this patient population. More research is needed in this area. However, studies to date suggest that hypofractionation with high doses as used for SBRT may worsen hypoxia in tumors,^45^ including NCSLC^46^ and result in reduced cell kill. The different biological response of tumors treated with hypofractionation of larger doses compared to conventional regimens may have resulted in the uncertainty that exists on the optimal prescription dose and number of treatment fractions that should be used for treating early‐stage NSCLC with SBRT.^32^, ^47^ As Ruggieri et al. point out, tumor responses to SBRT doses seem to be dominated by the response of the hypoxic cell subpopulation in the tumor, which may change the assumed tumor *α*/*β* ratios that are based on well‐oxygenated cells.^47^ Given that hypoxia is associated with poor prognosis, including for early‐stage NSCLC^48^, ^49^ and poor response to radiation therapy,^50^ this is an aspect that needs to be further explored and perhaps integrated into treatment planning before treating all early‐stage NSCLC tumors with SBRT.

On a final note, one of the most important factors that would support the use of lobectomy in early‐stage lung cancer patients is the prognostic information one would derive from lymph node dissection that is part of the lobectomy procedure. About 13%–32% of patients deemed negative for hilar/mediastinal lymph node involvement by positron emission tomography (PET) staging have been found to have lymph node involvement upon pathological evaluation.^51^ The “surprise” N1/N2 disease involvement is the indication for adjuvant chemotherapy and/or radiotherapy. Patients would not have this procedure done after SBRT. Although surgical staging of the mediastinum is encouraged in large academic centers, many patients would not be able to have such staging (endobronchial ultrasound‐guided fine‐needle aspiration biopsy or mediastinoscopy) due to a variety of reasons. This further underscores the need in exercising caution while deciding if SBRT can replace surgery in operable patients.

In conclusion, taking into account the information currently available through randomized clinical trials and the lack of long‐term survival data, the use of SBRT for all early‐stage patients is not justified, and surgery should remain the gold standard for patients without comorbidities who can tolerate surgery.^32^

## 4 REBUTTAL

### 4.A Pranshu Mohindra, MD; Amit Sawant, PhD; Robert Griffin, PhD

Both teams agree that for medically inoperable early‐stage NSCLC patients, SBRT should be the treatment of choice. Our disagreement is whether SBRT should also be recommended as the first option, rather than surgery, to operable patients.

In their opening statement, the opposing team bases their arguments on an almost literal interpretation of the word “all.” As clinicians and scientists, we can all agree that no medical intervention can claim applicability to “all” patients — there will always be exceptions. Setting aside this literal interpretation, the opposing team's position can be broken down into four parts — (a) lack of level‐1 evidence showing noninferiority/superiority of SBRT vs surgery, (b) high toxicity with SBRT when treating central/ultracentral lesions, (c) resistance of hypoxic tumors to SBRT, and (d) infeasibility of EBUS/FNA or mediastinoscopy‐based staging in SBRT. Upon closer examination, none of these arguments are convincing.

First, we agree with our opponents that evidence from randomized controlled trials (RCTs) is sorely needed. Most of the current debate surrounding this issue is guided by retrospective analyses. Our opponents acknowledge that these retrospective studies have significant limitations, the chief among them being selection bias. For example, in the 2016 NCDB analysis which showed superior overall survival (OS) with surgery, there were significant limitations including lack of cancer‐specific outcomes (local/regional/distant control), longer time from diagnosis to treatment in the SBRT cohort (72 vs 33 days, *P* < 0.001) and higher use of adjuvant chemotherapy in the lobectomy cohort (12% vs, 2%) either for pathological nodes (12%), larger tumors or positive margins.^18^ Indeed, among other studies cited by our opponents, with appropriate use of propensity score‐matching, SBRT showed performance similar or superior to surgery.^19^, ^20^ Further, salvage of a failure is always more challenging than treating adjuvantly, which confounds the analysis for SBRT nodal failures. Such SBRT eligible patients could benefit from more standardized staging such as EBUS + PET‐CT or adjuvant chemotherapy post‐SBRT for higher risk tumors (larger than 5 cm or central) as suggested in many experiences including a recent NCDB analysis.^52^ Notably, the only available data analysis to date from prospective randomized trials (pooled STARS/ROSEL) supports SBRT over surgery.^8^ At best, the evidence to date points toward equipoise between the two modalities. For these reasons, we believe that unless evidence from RCTs comes out unequivocally in favor of surgery, SBRT, due to its noninvasive nature and overall convenience to the patient, should be considered the primary treatment option rather than surgery.

Second, we disagree with our opponents’ statement “it is not safe to treat tumors located more centrally and close to critical organs.” To support their argument, our opponents cite a study from over 12 yr ago, showing increased toxicity for central tumors with a 3‐fraction regimen.^38^ To state the obvious, things have changed since then. Understanding and experience of respiratory motion management and associated motion management technology have vastly improved. Such improvements include routine use of 4D CT for simulation, advances in dose calculation algorithms and treatment plan optimization, routine use of cone‐beam CT for in‐room localization, and on‐line real‐time surrogate‐based or image‐based position monitoring for better spatiotemporal localization of the tumor with respect to surrounding OARs.^11^, ^53^ Prospective clinical trials such as RTOG 0813 have demonstrated in a phase −1/2 setting, the relative safety of using five fraction SBRT regimens for central and ultracentral tumors.^54^ Thus, while OAR risk is admittedly higher for central compared to peripheral tumors, it is increasingly no longer considered unacceptably high. It is also important to note that, when the option of surgery is considered, patients with central tumors will not be candidates for a sub‐lobar resection, leaving only the option of a lobectomy.

Third, we acknowledge our opposing team's contention that early‐stage NSCLC tumors may vary histologically, genetically and metabolically, potentially leading to heterogeneity in radiation response. However, we do not believe that this precludes the use of SBRT. The studies cited by our opponents to show the inefficacy of SBRT for hypoxic tumors do not provide compelling evidence to support their argument. For example, the modeling study by Carlson et al.^45^ uses a highly simplified model of capillaries vs tumor cells and does not model lung cancer (prostate and head and neck cancer). Moreover, our opponents overinterpret the study by Kelada et al., which is based on a relatively small patient cohort (N = 6).^46^ In contrast, a more recent modeling study by Jeong et al., using clinical data from 2701 early‐stage NSCLC patients, concluded that hypofractionated regimens such as SBRT overcome hypoxia and cell‐cycle radiosensitivity variations, as opposed to conventionally fractionated regimens. In the latter strategy, benefits of reoxygenation are negated by the prolonged treatment times, which allow for proliferation.^55^ Furthermore, there is a substantial body of clinical data that supports use of SBRT even for radioresistant tumors.^56^, ^57^ Novel strategies such as the Moffitt Cancer Center's approach for considering genomically adjusted radiation dose guided by radiosensitivity index are the likely direction for effective SBRT for all lung lesions.^58^

Finally, the rationale that EBUS/FNA or mediastinoscopy may not be feasible for radiotherapy patients is not a valid reason to preclude SBRT. If centers are able to do oncological lobectomy, there should not be any limitation in performing invasive mediastinal staging as part of standard of care.

In conclusion, there is mounting clinical evidence that SBRT is comparable if not superior to surgery in terms of efficacy and toxicity. We strongly anticipate that these findings from will soon be backed by results from ongoing RCTs.

### 4.B Narottam Lamichhane, PhD; Erina Vlashi, PhD; Meng Welliver, MD, PhD

We agree with Drs. Mohindra, Griffin and Sawant that lung SBRT is an upcoming, maturing treatment modality for patients with early‐stage NSCLC as an alternative to surgery. However, we still strongly believe that SBRT should remain an alternative to surgery, rather than replace the standard‐of‐care. Despite the promises that SBRT holds, as outlined by our colleagues in the “for the motion” opening statement, SBRT has not yet been demonstrated to be superior to surgery in well‐accrued, sufficiently powered, randomized clinical trials. As we outlined in our opening statement, there are compelling clinical and biological reasons that argue against treating all early‐stage NSCLCs with SBRT — we would like to re‐emphasize here in the closing statement that we stand against the word “all” in this motion.

It seems that the strongest argument for the use of SBRT in treating all early‐stage NSCLC with SBRT is the positive impact that it can have on the QoL and treatment convenience for these patients, undoubtedly very important considerations for patients who face invasive treatments. However, when comparing more unequivocal end points such as OS or disease specific survival (DSS), the jury is still out on which treatment modality is superior in matched patient populations. We strongly feel that with the data currently available it would be unwise to change clinical practice to treat all patients with SBRT, especially those patients who are medically operable. SBRT treatments are not without any toxicities. For example, fatal toxicities have been observed in patients with centrally located tumors after SBRT treatment.^38^ Additional challenges include the comparison of surgery to SBRT as it remains inherently difficult to control for the effect of comorbidities between the surgery‐eligible and non‐eligible patients.^59^ In a retrospective study, a matched comparison based on age, tumor size, location, and comorbidities showed that the 3‐yr OS was better in the surgery group compared to SBRT.^59^, ^60^, ^61^ Hence, while important insights have been gained from these studies, challenges remain with respect to determining the optimal dose–response relationship that results in improved survival with minimal toxicities, as well as in appropriate comparisons between the benefits of surgery vs SBRT in patients that differ in age, tumor size and location and other related comorbidities.^62^ Furthermore, studies also show that regardless of whether SBRT is a viable option for larger tumors, such tumors seem to be associated with more distant failures, thus needing extensive staging and adjuvant therapy.^63^, ^64^

As Drs. Mohindra, Griffin and Sawant pointed out, there is a surge of prospective clinical trials comparing the effectiveness of SBRT with surgery in NSCLCs. These trials are still accruing patients or have closed due to the lack of accrual. While compelling evidence exists, the data collected so far are inconclusive and await maturation. The conclusions from these trials are crucial and the field should await the results before changing the standard of care to offer SBRT to all early‐stage NSCLC patients purely based on QoL and convenience.

## CONFLICT OF INTEREST

The authors declare no conflicts of interest.
